# Long Lasting Persistence of *Bacillus thuringiensis* Subsp. *israelensis* (*Bti*) in Mosquito Natural Habitats

**DOI:** 10.1371/journal.pone.0003432

**Published:** 2008-10-20

**Authors:** Mathieu Tilquin, Margot Paris, Stéphane Reynaud, Laurence Despres, Patrick Ravanel, Roberto A. Geremia, Jérôme Gury

**Affiliations:** 1 Floralis-UJF Filiale, Gières, France; 2 Laboratoire d'Ecologie Alpine (LECA), CNRS UMR 5553, Universite' Joseph Fourier, Grenoble, France; Centre for DNA Fingerprinting and Diagnostics, India

## Abstract

**Background:**

The detrimental effects of chemical insecticides on the environment and human health have lead to the call for biological alternatives. Today, one of the most promising solutions is the use of spray formulations based on *Bacillus thuringiensis* subsp. *israelensis* (*Bti*) in insect control programs. As a result, the amounts of *Bti* spread in the environment are expected to increase worldwide, whilst the common belief that commercial *Bti* is easily cleared from the ecosystem has not yet been clearly established.

**Methodology/Main Findings:**

In this study, we aimed to determine the nature and origin of the high toxicity toward mosquito larvae found in decaying leaf litter collected in several natural mosquito breeding sites in the Rhône-Alpes region. From the toxic fraction of the leaf litter, we isolated *B. cereus*-like bacteria that were further characterized as *B. thuringiensis* subsp. *israelensis* using PCR amplification of specific toxin genes. Immunological analysis of these *Bti* strains showed that they belong to the H14 group. We finally used amplified length polymorphism (AFLP) markers to show that the strains isolated from the leaf litter were closely related to those present in the commercial insecticide used for field application, and differed from natural worldwide genotypes.

**Conclusions/Significance:**

Our results raise the issue of the persistence, potential proliferation and environmental accumulation of human-spread *Bti* in natural mosquito habitats. Such *Bti* environmental persistence may lengthen the exposure time of insects to this bio-insecticide, thereby increasing the risk of resistance acquisition in target insects, and of a negative impact on non-target insects.

## Introduction

Since the fifties, the massive use of chemical insecticides in insect control programs, although very effective in most cases, has led to serious environmental problems, including the long-term persistence of the toxicity in the environment, leading to the acquisition of resistance in exposed insects [1]. Over the past decades, the trend has been towards a reduction in the use of chemical insecticides, progressively replaced by emerging environment-friendly pesticides such as bacterio-insecticides, strongly recommended by the World Health Organization [2].

*Bacillus thuringiensis* subsp. *israelensis* (*Bti*) is one of the most famous spore forming bacterium, able to produce specific insecticidal toxins during sporulation [3]. The toxin is a combination of six main proteins aggregated into a solid crystal encased in the bacterial cell [4], exhibiting acute toxicity towards dipteran insects such as larval mosquitoes and black flies. Today, this bacterium is widely used for the preparation of commercial bio-insecticides [5] used in insect control programs.

The main advantage of *Bti* over chemical insecticides is its highly specific activity towards dipteran insects, due to the presence of membrane receptors in the insect gut serving as targets for the bacterial toxins [3], [6], [7]. Due to the absence of such receptors in vertebrates, the *Bti* is considered safe for human health [8]. It was claimed that the toxins and spores were not persistent in the environment with virtually no residual effects, even in environments submitted to seasonal applications [3], [9]. Furthermore, almost no dispersion of the spores was observed in the soil [10]–[12], and contamination of ground water seems very unlikely 13, 14. Finally, due to the complex structure of *Bti* toxins, many authors emphasized that the acquisition of resistance in exposed insects would require multiple mutations at different loci, and is therefore largely delayed under natural conditions [15]–[17].

For these reasons, the use of *Bacillus thuringiensis* based insecticides in pest control programs is now considered as a viable strategy, which has proven to be both safe and reliable over the last 40 years [18]–[20].

The growing importance of bacterio-insecticides in insect control activities has encouraged many research programs aiming to discover new bacterial strains with improved insecticidal properties.

In 2000, David et al. reported the presence of highly toxic leaf litter in forest mosquito breeding sites [21]. Due to the acute lethal effects measured on larval *Aedes aegypti*, and the urgent need for new insecticidal molecules, considerable efforts were made to isolate and identify the compounds responsible for the leaf litter toxicity [22]–[25]. This led to the development of a laboratory process for synthesizing toxicity from non-toxic decomposed leaf litter. In this article we present the molecular and immunological proof that the high toxicity of leaf litter relies on the persistence of *Bti* spores in natural mosquito habitats. Furthermore, the presence of *Bti* viable spores in untreated areas raises concerns regarding the ecological consequences of massive bacterio-insecticides spreading on a regional scale.

## Results

### Identification of Bti in toxic and non-toxic leaf litter

**Figure 1:**
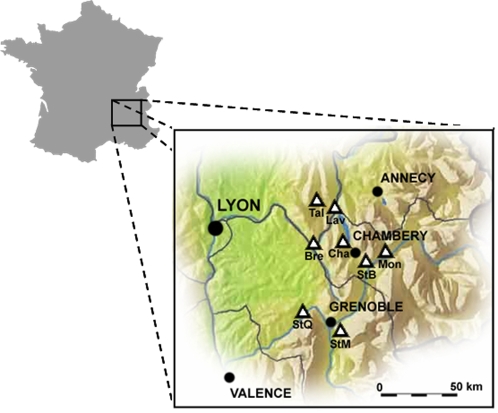
Map of sampling sites in the Rhône-Alps region (France). Each triangle corresponds to a sampling area. All of them are humid mosquito breeding areas (natural forests or swamps) except for the StM site, which is an artificial urban site intended for leaves storage.

Different leaf litter samples were tested for *in vitro* reconstitution of the toxicity (table 1 and Figure 1). None of these leaf litter samples were toxic *per se*, however all of them produced toxicity *in vitro* after the laboratory process described in table 2. The vegetation type did not seem to have an influence on toxicity production, neither did the year of collection. Samples originating from both treated and untreated areas were able to generate toxicity *in vitro*. The insecticidal efficacy of the toxin synthesized during the *in vitro* process was measured in bioassays, using larvae of the mosquito *Aedes aegypti* as a standard organism. An LC_50_ of 0.02 mg l^−1^ was obtained for this toxin, comparable to those for *Bti* [26].

**Table 1:** description of the eight sampling sites where decaying leaf litter were collected.

Site	Type	Occurrence of treatment^a^	Dominant species	Year of sampling	Toxicity in vitro^b^
Tal (1)	Forest	treated	*Alnus glutinosa*, *Fraxinus excelsior*	2002, 2003, 2005	+
Lav (2)	Forest	treated	*Alnus glutinosa*, *Fraxinus excelsior*	2002, 2003, 2005	+
Bre (3)	Forest	treated	*Alnus glutinosa*, *Quercus robur*	2002, 2003	+
Mon (4)	Forest	treated	*Alnus glutinosa*, *Alnus incana*	2002	+
StQ (5)	Forest	treated	*Alnus glutinosa*	2002	+
StB (6)	Forest	untreated	*Alnus glutinosa*, *Fraxinus excelsior*	2006	+
Cha (7)	Swamp	treated	*Carex sp.*	2002	+
StM (8)	Artificial	untreated	*Populus nigra*	2005, 2006	+

a The sites referred to as “treated” correspond to mosquito breeding sites where annual spreading of the insecticide *Bti* occurred for more than ten years. No *Bti* treatment was ever applied in “untreated” areas.

b The leaf litter samples were submitted to the 3-step process for *in vitro* synthesis of the toxicity: when more than 80% mortality was obtained in the bioassays for at least two leaf litter samples from the same site, the columns were referred to as “+”.

**Table 2:** Experimental conditions for the in vitro synthesis of toxicity.

Treatment	Modality	Toxicity^a^
	without	−
Heat shock (10 min)	70°C	+
	100°C	−
Sterilization	with	−
(0.2 µm filtration)	without	+
Incubation (3 days)	25°C	+
	5°C	−

a Different treatment modalities were tested on the leaf litter extract and the toxicity produced under these conditions was measured by standard bioassays on *Aedes aegypti* larvae (at least 80% mortality: marked “+”; under 5% mortality: marked “−”).

In order to better understand the mechanisms involved in the *in vitro* synthesis of toxicity, different experimental conditions were tested. Those with the most significant effect on the production of toxicity are summarized in table 2. Heat shock (70°C) was necessary to obtain toxicity whereas 100°C or 0.22 µm filtration treatments prevented it. The last step of the process consists in incubating the extract at 25°C over 72 h. The extract becomes turbid and a toxic pellet is formed. All these results suggest that a micro-organism is involved in leaf litter toxicity.

**Figure 2:**
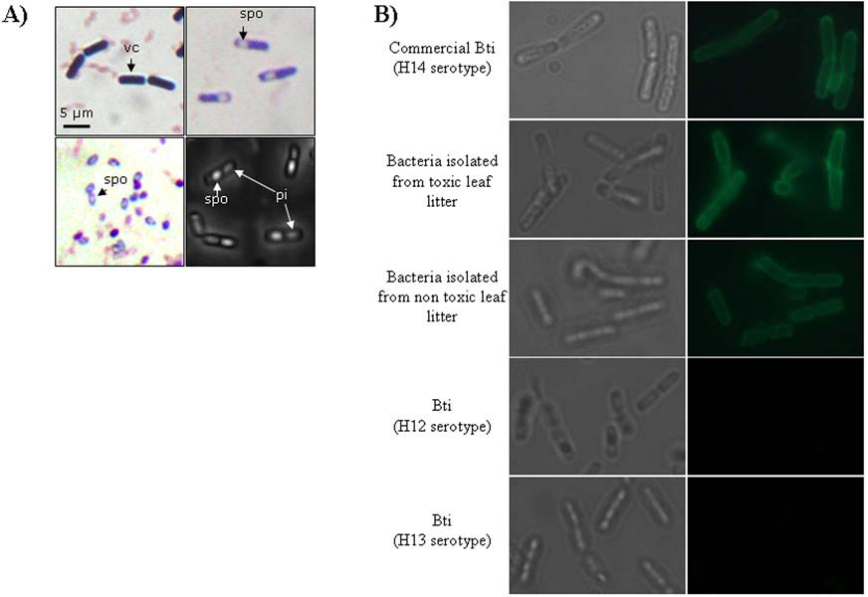
Microscopic observations of different *Bacillus thuringiensis* subsp. *israelensis* strains. A) Oil immersion microscopy observation of the vegetative cells (vc) and spores (spo) after Gram staining of isoloated toxic bacterium of leaf litter. The parasporal inclusions (pi) were observed by phase contrast microscopy. B) Fluorescent microscopy serotype analysis of *Bti* isolated from toxic or non toxic leaf litter. Bacilli were stained with rabbit antiserum specific for flagellar H14 serotype. Rabbit antibodies were revealed with FITC-conjugated goat anti rabbit Ig.

The presence of microorganisms was investigated by plating aliquots of the leaf litter extracts on LB agar plates. From the different isolated bacteria, it was possible to synthesize toxicity when grown in either LB medium, or sterile leaf litter extract medium. The involvement of this bacterium in producing toxicity was confirmed by its presence in all leaf litter samples previously identified as “toxicity producing”. This bacterium is 5 µm long, Gram-positive, rod-shaped and spore-forming (Figure 2A). The phase-contrast microscopy revealed the presence of a parasporal inclusion in sporulating cells. This microscopic evidence together with the insecticidal capacity of the bacteria, matched the criteria for the *Bacillus cereus* group. The affiliation to the *Bacillus cereus* group was further confirmed by the sequencing of the 16 s RNA gene, resulting in 100% homology with 16S RNA sequences available in GenBank for this species group (data not shown). The complete determination was adapted from Ben-Dov et al. [27] by using specific primers for different toxin-coding genes (table 3). The strain was positive for six genes, namely *cyt1*, *cyt2*, *cry4A* and *B*, *cry10* and *cry11* respectively. According to the literature, this combination of genes is typically found in the bacteria *Bacillus thuringiensis* subsp. *israelensis*, and the absence of *cry1* and *cry8* genes (serving as controls) supported the determination (see Figure S1). In addition, the 300 bp Cry11 PCR fragment was sequenced and resulted in a 100% match with *Bacillus thuringiensis israelensis* (GeneId: 5759849, [28]). A final identification method based on immunofluorescence with antibodies specific to flagellar proteins was used to determine the serotype of the isolated strains. The bacteria isolated from commercial *Bti* known to be of the H14 serotype, were marked with fluorescence (green) whilst no reaction was observed for closely-related serotypes such as H12 and H13 (Figure 2B). This result confirmed the specificity of the flagellar antibody and therefore, the fluorescence observed for the bacteria isolated from toxic and non-toxic leaf litter made it possible to establish them as having an H14 serotype.

**Table 3:** Primers used for PCR amplification.

Primer pair	Anneal temp (°C)	Sequence^a^	Reference
1492r (16 s RNA)	50	5′ TACGGTTACCTTGTTACGACTT (r)	[57]
27f (16 s RNA)	50	5′ AGAGTTTGATCMTGGCTCAG (d)	[57]
cry1gral	52	5′ CTGGATTTACAGGTGGGGATAT (d),	[58]
		5′ TGAGTCGCTTCGCATATTTGACT (r)	
cyt1gral	52	5′ CCTCAATCAACAGCAAGGGTTATT (d),	[59]
		5′ TGCAAACAGGACATTGTATGTGTAATT (r)	
cyt2gral	50	5′ ATTACAAATTGCAAATGGTATTCC (d),	[59]
		5′ TTTCAACATCCACAGTAATTTCAAATGC (r)	
cry4Aspe	50	5′ TCAAAGATCATTTCAAAATTACATG (d)	[59]
cry4Bspe		5′ GTTTTCAAGACCTAATAATATAATACC (d),	
		5′ CGGCTTGATCTATGTCATAATCTGT (r)	[59]
cry8gral	49	5′ ATGAGTCCAAATAATCTAAATG (d),	[58]
		5′ TTTGATTAATGAGTTCTTCCACTCG (r)	
cry10spe	51	5′ TCAATGCTCCATCCAATG (d),	[59]
		5′ CTTGTATAGGCCTTCCTCCG (r)	
cry11gral	51	5′ TTAGAAGATACGCCAGATCAAGC (d),	[58]
		5′ CATTTGTACTTGAAGTTGTAATCCC (r)	

a d and r, direct and reverse primers, respectively.

**Figure 3:**
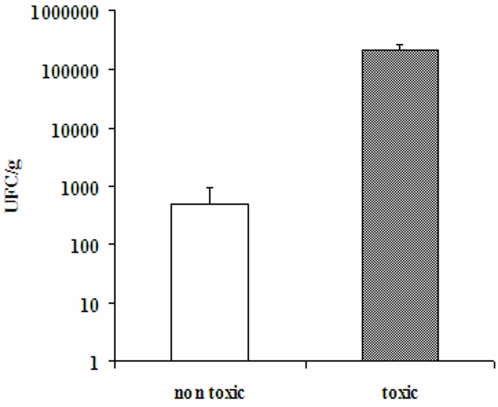
Counts in leaf litter samples of viable spores of *Bacillus thuringiensis* subsp. *israelensis* expressed as number of UFC per gram of dry matter (mean±SE, log scale). The bars correspond to the standard error calculated from six samples of non-toxic leaf litter and three samples of toxic leaf litter.

Besides the non-toxic leaf litter, we focused on particular leaf litter samples collected at one of our sampling sites in 1998, which were highly toxic *per se* (referred to as “toxic leaf litter”). Figure 3 shows the number of viable spores of *Bti* per gram of dry matter, counted in either non-toxic (6 samples) or toxic (3 samples) leaf litter samples. The mean number of viable spores in toxic leaf litter was about 400 times that in non-toxic samples. Depending on the sample, up to 300,000 spores per gram were counted in toxic leaf litter, indicating a high bacterial load for these samples.

Of all the colonies isolated from toxic leaf litter, PCR amplifications revealed that more than 90% could be identified as *Bti* (positive to *cyt1* and *2*,*cry4A* and *4B*, *cry10* and *cry11* genes, data not shown).

**Figure 4:**
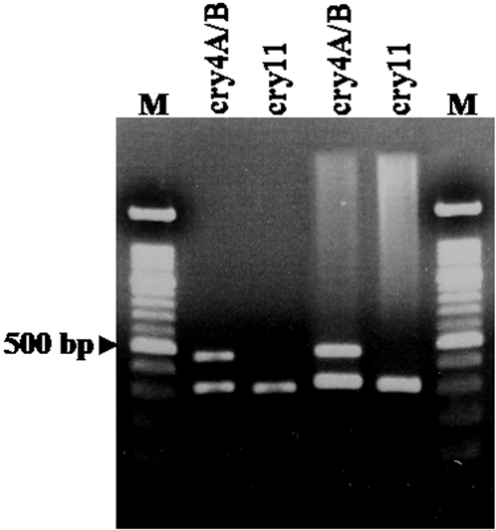
Agarose gel electrophoresis of the PCR products obtained with the DNA extracted from toxic leaf litter. The total DNA was extracted and purified from toxic leaf litter samples and the *cry 4A*, *cry4B* and *cry11* genes were sought by PCR using the corresponding primers. The PCR products detected from toxic leaf litter (lanes 2 and 3) were compared to those obtained from the isolated bacterium serving as a control (lanes 4 and 5).

We also investigated using a more direct method for detecting the bacteria in field collected leaf litter that does not require the isolation of the bacteria on agar plates. The genes encoding *Bti* toxins Cry4A, 4B and Cry11 were directly sought by PCR in the total DNA pool extracted and purified from leaf litter material (Figure 4). For toxic leaf litter, only one run of 31 PCR amplification cycles was sufficient for *Bti* diagnostic, whereas two runs were required to amplify diagnostic *Bti* genes from non toxic litter.

### Origin of the *Bti* found in natural habitats

Some of the sampled sites were regularly sprayed with commercial *Bti*, by the local mosquito control agency, raising the question of the origin of the *Bti* strains found in leaf litter (natural vs human-spread). A total of 201 polymorphic markers ranging in size from 67 to 522 pb were successfully scored with the two primer combinations used. The genotyping error rate per locus has been calculated as the ratio between the observed number of differences and the total number of comparisons across the duplicated samples [29]. The seven duplicates were highly reproducible (99.85%). Among the 23 strains genotyped, the toxic litter sample StM and the commercial strain WG1 presented the same AFLP profiles. All the others strains presented different profiles. Among the *Bti* H14 strains, 52 out of 201 markers (25.8%) were polymorphic. Eighteen markers were diagnostic for *B. sphaericus* strains (i.e. markers always detected in *Bs* and never in *Bti* strains) and eight for *Bti* strains. No diagnostic marker was associated with *Bti* H14 leaf litter strains, or *Bti* H14 commercial strains, or *Bti* H14 natural worldwide strains.

Pairwise Jaccard distance coefficients among strains varied from 0 (Tal1 vs. WG2) to 0.95 (S05 vs. S05_201) with an overall mean of 0.58±0.29. Interspecific average distance (*B. shaericus* vs. *Bti*) was 0.92±0.01. For the *Bti* strains, a mean distance of 0.81±0.03 was found between different serotypes, and a mean distance of 0.29±0.10 was found between strains within the H14 serotype. Within the H14 group, the mean distance was 0.17±0.07 between commercial and leaf litter strains, 0.33±0.8 between natural and leaf litter strains, and 0.32±0.8 between natural and commercial strains.

**Figure 5:**
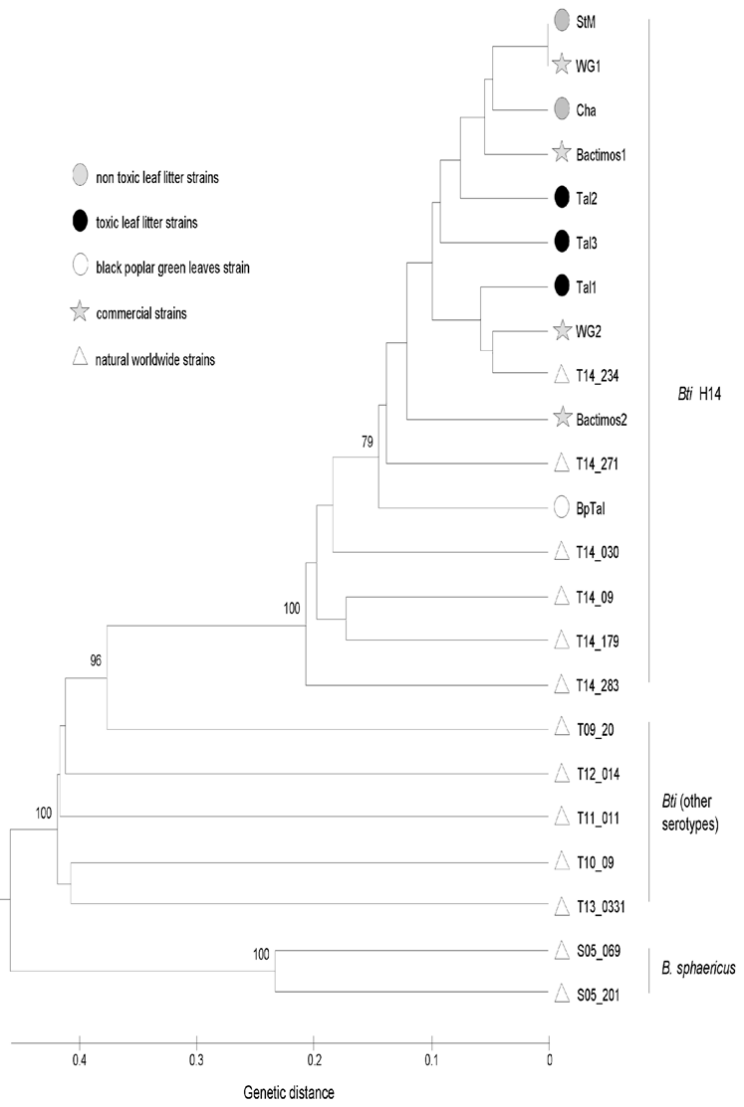
Dendrogram based on the AFLP profiles (201 polymorphic markers) from 21 *Bacillus thuringiensis* subsp. *israelensis* strains (6 serovars) and 2 *Bacillus sphericus* strains. Genetic distance between AFLP profiles was calculated by using Jaccard distance. Bootstraped data set were generated using PHYLTOOLS (1,000 replicates) and analyzed by using the UPGMA clustering method (PHYLIP package V3.5). Only bootstrap values more than 70% are represented.

The UPGMA dendrogram based on Jaccard distance clearly grouped all the H14 serotype strains together (Figure 5). Within this group, all the strains isolated from the commercial *Bti* were intermixed with strains isolated from toxic and non-toxic litter, and there was one natural strain from the IP collection collected in 1991 in Nigeria. All the other natural *Bti* strains were more distant. Analysis of molecular variance showed a significant genetic differentiation between the group encompassing all the commercial strains and the non-toxic and toxic litter, and the group containing all the worldwide natural strains and the strain isolated from green leaves at a Rhône-Alps site (*P*<0.0001). It therefore appears that *Bti* isolated from green leaves is more closely related to natural worldwide strains than to commercial *Bti*.

## Discussion

*Bti* belongs to the *Bacillus thuringiensis* species that is usually isolated from the soil but can also be found in many other habitats such like insects (where it proliferates), stored grains, and the phylloplane of plants [30]–[32] although it was described as a poor colonist of leaf surfaces [33].

Contrary to our observations, *Bti* is generally absent from untreated areas [33]. Some authors excluded the possibility of *Bti* accumulation in treated areas, even when submitted to pluri-annual treatments [34], [35] Actually, even if they were able to persist in soils, the spores of *Bti* have never been seen to germinate and proliferate in this compartment where, on the contrary, they tend to be gradually inactivated [34], [36]. In our study, not only was *Bti* found in two untreated sites, but it was also detected at high levels in some samples associated with an acute toxicity towards mosquito larvae. The high *Bti* content, estimated by the number of viable spores, appeared to strongly correlate to the toxicity of the leaf litter samples. To the best of our knowledge, no reference to such residual toxicity in relation to *Bti* accumulation has been made in the literature so far.

The originality of our work lies in the type of samples analyzed. Whereas most of the studies addressing the question of *Bti* occurrence in the environment deal with soil or water samples, we focused on decaying leaf litter, the natural habitat of mosquito larvae. In light of our results, it seems that decaying leaves represent a specific microhabitat in which *Bti* is more likely to persist and potentially grow than in soils. The spores and toxins may be protected from degradation by the vegetal matrix, which makes accumulation processes possible. Although unusual, the germination of *Bacilli* spores in microhabitats other than soils has already been suggested. So far, even if able to persist in soils, the spores of *Bti* were never observed to germinate and proliferate in this compartment where, on the contrary, they tend to be gradually inactivated [34] especially in an environment with high organic matter content. Since the site where the toxic leaf litter was collected was submitted to a single *Bti* treatment during the year 1998, the elevated number of spores (300,000 spores per gram of dry matter) is unlikely to be due to accumulation only. The possibility that the bacteria germinate and proliferate in the natural habitat must not be excluded. Indeed, the germination of spores of *B. thuringiensis* subsp. *kurstaki* was detected in natural conditions, in the rhizosphere and in the gut of insects collected in their habitat [37]. The question arises about the biotic and abiotic conditions that allowed persistence and proliferation of *Bti* in this particular breeding site in comparison with the others. Further investigations would be necessary to fully characterize sites factors such as soil composition, pH, temperature, ultraviolet exposition and insect diversity. In a first attempt to characterize the abiotic conditions of the Rhône-Alps mosquito breeding sites, our investigations on the vegetation and the soil revealed lower oxygen levels in the site where toxic material was collected (Tilquin M., unpublished results). A study of the leaf litter decomposition rates over a year confirmed this observation, and showed that the processing of organic matter was significantly slower in the “toxic site”, causing the leaves to remain on the ground for a longer time period (data not shown). These observations suggest that under these particular conditions (low oxygen level and low leaf litter decomposition rate), the *Bti* had more time to colonize the leaf litter material, or/and was less affected by degradation than in the other sites.

### Tools and methods for the study of bacterio-insecticides in the environment

In this work, we have adapted several tools that have proved to be reliable and to offer interesting opportunities to study of the fate and behavior of bacterio-insecticides in the environment.

Our selective laboratory protocol in conjunction with toxicological bioassays on mosquitoes allows the detection of bacteria exhibiting insecticidal properties.

In addition to its simplicity, the main interest of the protocol lies in the selection of spore-forming organisms by means of the heat shock treatment, considerably reducing the number of microorganisms to be studied. The possibility of selecting interesting candidates among the great diversity of microorganisms encountered in natural substrates such as leaf litter or soil appears to be a valuable advantage. This protocol could therefore be used as an efficient tool to search for the compartments of the ecosystem where bacteria exhibiting insecticidal properties are present, even at low levels.

In addition to the toxicological approach, microscopic and molecular cues can also be obtained from this protocol. As shown in this study, the presence of *Bacillus cereus*-like (*Bc*-like) colonies is easily revealed by plating the heat-shocked extracts on agar plates.

Once isolated on a solid medium, the counting of *Bc*-like colonies appears to be a reproducible method for the quantification of the bacterial population from our samples. In our case, substantial differences in terms of bacterial load were observed between toxic and non-toxic leaf litter.

The microscopic observations of the colonies, revealing the presence of spores and parasporal inclusions in the vegetative cells, supported our toxicological results.

Finally, the PCR amplification of *cyt* and *cry* genes, coupled with immunoassays with flagellar antibodies, clearly identified the isolated organisms, up to the strain level.

The AFLP fingerprinting method has already proven to be a useful tool in bacterial phylogenetics [38], [39], species identification [40], [41] or epidemiologic studies [42], [43]. AFLP was also used successfully to detect molecular variability between highly closely related bacterial strains [44]–[46]. If previous studies analysed the diversity between different *B. thuringiensis* subspecies [47], to our knowledge, no comparative analyses have been performed between strains within the subspecies *israelensis* so far. In this study, AFLP revealed high polymorphism and allowed to easily discriminate six *Bti* serotypes.

In addition, sufficient genetic diversity was detected between the *Bti* strains within H14 serotype to distinguish between commercial and natural strains, and to identify the origin of leaf litter *Bti*. As AFLP is highly reproducible and highly discriminatory at the strain level, it could be the method of choice for the molecular characterization of *Bti* strains.

This type of multi-step method can easily be used to study *Bti* spread by man in various natural substrates, and to screen new strains of insecticidal bacteria.

Another interesting result of this work is the possibility of detecting bacterial DNA directly from the vegetal sample, without a prior isolation of the bacteria on agar plate (Figure 4). By reducing the number of laboratory stages required, and allowing the use of high throughput tools for the detection of the target micro-organism, this simple protocol provides interesting perspectives for the study of bacterio-insecticides in the environment. Furthermore, our results have shown that this method is sensitive enough to reveal quantitative differences between samples. We are currently adapting the quantitative PCR method to the detection and precise quantification of bacterial DNA in leaf litter samples. Bioassays and molecular detection by PCR represent an alternative method to western blot and ELISA techniques. Immunodetection requires *Bti* toxin purification and specific antibodies. These methods are time consuming and not suited for high-throughput environmental sample analyses, as the solubilization and purification of toxins from leaf litter of various origins is likely to be difficult to standardize.

### The anthropogenic origin of the Bti found in natural habitats

AFLP data obtained with the two selective primer combinations was high quality and revealed sufficient levels of polymorphism for non-ambiguous species and serotype identification, and for strain comparisons within the serotype H14. Indeed, the high level of genetic diversity observed between *Bti* strains was essential to effectively characterize the origin of sampled strains.

Although the *B. cereus* group is described as taxonomically confusing, with no phylogenetic differentiation between *B. thuringiensis* and *B. cereus* at the species level [38], [45], our AFLP data showed unambiguous differentiation between the 2 species *B. sphaericus* and *B. thuringiensis*. The clustering analyses also indicated a clear phylogenetic signal at the serotype level within the subspecies *B. thuringiensis* subsp. *israelensis*.

All the commercial and Pasteur Institute collection H14 *Bti* strains are grouped in a same cluster, indicating good discrimination of the serotype status of *Bti* strains using AFLP methods. The Rhone-Alps strains also grouped in the H14 *Bti* cluster, confirming the toxicological and immunological results. All the toxic and non toxic leaf litter strains were more closely-related to commercial strain than to worldwide natural strains. In addition, a non toxic leaf litter strain presented the same AFLP profiles as the Vectobac WG *Bti* commercial strain used in the Rhone-Alps region. These results strongly support the anthropogenic origin of the *Bti* found in treated and untreated areas, i.e., that spraying residues can persist and disseminate out from the spraying zone.

The presence of *Bti* strains that are genetically undistinguishable from commercial *Bti* strains at sites which have never been treated is a remarkable and unexpected result, and a wider sampling design would be required to confirm this finding. The very sensitive and reproducible methods developed in our work will be of great use for such wide-scale monitoring of treated and untreated sites on a regional scale.

### Conclusion

These results support previous work showing that the persistence of acute toxicity several months after *Bti* spraying are exceptional, as this was only observed on one of the eight sites analyzed. However, we identified the presence of viable *Bti* spores at all the other sites, including one site that had never been treated, suggesting that *Bti* regular spraying of some target sites may have a greater impact than previously thought on regional scale (Rhône-Alps region, about 3000 km^2^). The impact of such uncontrolled *Bti* residue on natural insect populations is a cause for concern, especially since leaf litter represents the main source of food for mosquito larvae. In case of *Bti*-persitence in leaf litter, mosquito larvae are exposed to *Bti* toxins throughout their whole development, even at low doses. Such repeated exposition can be considered as a typical selection pressure promoting resistance emergence in mosquito populations. Indeed, a previous study showed that mortality of *Aedes rusticus* to toxic-leaf-litter (containing *Bti*) was lower for larvae sampled in sites which were *Bti*-treated for more than 2 years [48] as compared to untreated sites. Furthermore, a recent article reported significant differences in the sensitivity of mosquito populations to insecticides in relation to *Bti* applications in wetland Rhône-Alps areas [49]. Given the increasing importance of bacterio-insecticides in general, and *Bti* in particular, in insect control programs, there is an urgent need to compile data on the fate and behavior of bacterio-insecticides in the environment. In this work, we highlighted some potential disorders in relation to *Bti* spreading, and we are currently focusing our research on the improvement of the methodology to ensure the rapid and accurate quantification of bacterial DNA in leaf litter samples. Our aim is to initiate a larger sampling campaign to monitor the behavior of *Bti* populations in different conditions for the application of insecticide, and different ecological situations.

## Materials and Methods

### Sampling sites and leaf litter collection

The leaf litter were collected between July and August 4 to 10 weeks after *Bti* treatment in eight sites in the Rhône-Alps region (Figure 1) between 2002 and 2006. At each location, ten samples of leaf litter (approximately 100 g of fresh matter) were randomly collected in the organic layer (OL). The samples were air dried at 30°C, powdered on a 0.5 mm mesh, carefully homogenized, and stored at −80°C in individual sterile flasks until use. All the samples were compared for their mosquito larvicidal activities to a toxic *per-se* leaf-litter [50]. This toxic litter was collected in August 1998 (site Tal), 4 months after a single *Bti* treatment application at the beginning of spring. In this particular site, a monthly increase (from spring to the end of summer) of leaf litter toxicity was observed for several consecutive years (1996–1998) [50].

### The three-step process for the synthesis of toxicity

Each leaf litter sample was used for the *in vitro* synthesis of the toxicity in the 3-step process developed by Tilquin et *al* [22]. Briefly, 200 mg of powdered leaf litter was stirred into 40 ml of sterile Na/K phosphate buffer (25 mM, pH 7.5) for 1 hr at 25°C (step 1: extraction). The leaf litter residue was discarded by filtration through Whatman # 2 after a short centrifugation (5 000 rpm for 5 minutes), and the collected extract was immediately heat shocked for 30 minutes at 70°C (step 2: heat shock), followed by centrifugation to remove all solid residue (15 min at 5 000 rpm). The collected soluble extract was incubated for 72 hrs at 25°C in sterile glass vials (step 3: incubation). During the incubation step, an insoluble fraction appeared in the extract. This precipitate was collected at the end of the incubation by centrifugation (30 min at 5 000 rpm), water rinsed with 5 ml of distilled water (re-suspended and centrifuged 5 min at 5 000 rpm) and immediately used for toxicological bioassays. All extractions were performed in triplicate.

### Toxicological bioassays

The toxicity of the precipitate was tested using a standard bioassay procedure on *Aedes aegypti* mosquito larvae as a reference [51]. This bioassay used 20 size-calibrated fourth instar larvae kept in a disposable vial containing 50 ml of tap water at 25°C. The precipitate collected after the third step of the synthesis process was re-suspended in 6 ml of distilled water by pipetting up and down, and the larvae were dosed with 2 ml of suspension. Each bioassay was performed in triplicate. Larval mortality was counted after two hrs of contact, and the toxicity of the tested material was expressed in % mortality.

The same bioassay protocol was used to determine the LC_50_ of the precipitate (i.e. concentration generating 50% mortality in the bioassays). An important amount of toxic precipitate was prepared by standard extractions (as described above) and re-suspended in distilled water. An aliquot of the suspension was filtered on a 0.22 µm Whatman filter previously weighed on a precision balance. The filter was then dried until no mass loss was observed, and weighed again to determine the mass of the precipitate. This value was then used to calculate de concentration of the suspension, and to prepare the bioassay media at different concentrations (ranging from 0.001 to 1 mg/l). The bioassays were conducted at 25°C in triplicate, and larval mortality was monitored after 24 hrs contact with insecticide and further analyzed using the Probit software to determine the mean LC_50_ [52].

### Bacterial isolation

#### Leaf litter microorganisms

The presence of microorganisms in the leaf litter and poplar leaf extracts was showed by plating 200 µl of extract on Luria Bertani (LB) agar plates immediately after step 2 of the synthesis process (heat shock). The plates were incubated for 24 hrs at 30°C to ensure bacterial growth. We estimated the bacterial load of leaf litter samples by counting the number of colonies (Colony-Forming Unit) per initial gram of dry leaf matter (40 ml of initial extract corresponded to 200 mg of dry leaf litter; 200 µl plated on agar corresponded to 1 mg of dry leaf litter). In order to test for their insecticidal properties, the colonies isolated on agar plates were introduced into sterile vials containing 40 ml of liquid LB broth, and grown at 25°C, without stirring. The cultures were centrifuged 72 hrs after sowing (5 000 rpm for 30 minutes), and the precipitates were re-suspended in 6 ml of distilled water to check for toxicity. The bioassays were performed according to the same protocol as previously described, with 2 ml of suspension per 50 ml of bioassay, in triplicate.

### DNA extraction and PCR amplification

Different molecular methods were used in this study to further identify the bacterial strains isolated from our leaf litter samples.

Bacterial DNA was extracted with Fast DNA spin Kit (MP Biomedicals, Illkirch, France) according to the manufacturer's recommendations. Leaf litter DNA was extracted with Powersoil DNA extraction kits (Mo Bio Laboratories, USA) according to the manufacturer's recommendations. The DNA concentrations were measured using a Nanodrop ND 1000 Spectrophotometer (Nanodrop Technology, USA).

The genes encoding for the toxic crystal proteins of the *Bacillus thuringiensis*, namely *cyt1* and *2*, *cry1*, *cry4a* and *b*, *cry8*, *cry10* and *cry11*, and 16 s RNA were sought by PCR amplification using either primers described in table 3.

Bacterial DNA was extracted using a Fast DNA spin Kit (MP Biomedicals, Illkirch, France) according to the manufacturer's recommendations. Leaf litter DNA was extracted with Powersoil DNA extraction kits (Mo Bio Laboratories, USA) according to the manufacturer's recommendations. The DNA concentrations were measured using a Nanodrop ND 1000 Spectrophotometer (Nanodrop Technology, USA).

### Serotype characterization

For the flagellar serotype analysis of the *Bti* isolated from toxic or non toxic leaf litter, bacilli were incubated with rabbit antiserum specific for H14 serotype (provided by the Pasteur Institute, France) during 15 minutes at room temperature. After 3 washes with PBS, bacilli were incubated with FITC-conjugated goat anti-rabbit Ig (Southern biotech, USA), on slides, and serotype analysis was done using fluorescent microscopy (BX41 Olympus, France). Control strains (i.e. H12 and H13 serotypes) were obtained from the Pasteur Institute's collection (Paris, France), and the H14 serotype was isolated from the commercial *Bti* formulation (Vectobac WG, Valent Biosciences Corp.). In both cases, the strains were cultivated on LB agar medium at 30°C. Prior to microscopic observation, the gram staining method was used.

### AFLP genotyping

*Bacillus* strains used for the AFLP genotyping are listed in Table S1: 13 natural worldwide strains were obtained from the Pasteur Institute's collection, 4 commercial strains were isolated from Bactimos and Vectobac WG *Bti* commercial formulations used as spray insecticides in the Rhône-Alps region, and 6 strains were isolated from leaf litter or green leaves collected in various sites in the Rhône-Alps region.

Total genomic DNA was extracted from overnight culture at 27°C of control and isolated bacterial strains with the DNeasy tissue Kit (Qiagen) following the Gram positive bacteria protocol. DNA concentration and quality was determined by spectrometry on a NanoDrop and each sample was diluted to 30 ng/µl for the AFLP analyses [53].

Genomic DNA was first digested with *Mse*I (150 ng of DNA, 1× of Buffer NEB2, 1 µg of BSA and 2 U of *Mse*I in a 17 µl reaction) and then with *Eco*RI (1× of Buffer *Eco*RI and 5 U of *EcoR*I in a 27 µl reaction). Specific oligonucleotidic adaptors were then ligated to the end of the restriction fragments (T4 DNA ligase buffer 1×, 10 pmol of *EcoR*I adaptator, 50 pmol of *Mse*I adaptator, and 1 U of T4 DNA ligase in a 37 µl reaction). The restriction and ligation reactions were incubated at 37°C for 2 h30.

Selective amplification was performed as previously described [54]. PCR parameters were: 10 min at 95°C followed by 36 cycles of 30 s denaturing at 94°C, 30 s annealing, and 1 min extension at 72°C, ending with 10 min at 72°C for complete extension. Annealing was initiated at a temperature of 65°C, which was reduced by 0.7°C for each of the next 12 cycles and maintained at 56°C for the subsequent 23 cycles. Two selective PCR primer pairs were selected over 8 tested for the quality of the produced bands (i.e. even distribution of bands with relatively homogeneous intensity): E+A/M+C and E+A/M+G. Products were separated by electrophoresis on an ABI 3130xl capillary sequencer. AFLP patterns were then visualised with GenMapper V3.7 software: a fluorescent peak corresponds to the presence of an amplified restriction fragment. Polymorphic peaks were checked individually and a presence/absence (i.e 1/0) matrix was constructed. Reproducibility of each primer pair was checked by carrying out two times the whole AFLP protocol for 30% of individuals randomly chosen as recommended by Bonin et al. (2004) [55].

#### Genetic analysis

Pairwise distances were determined by the Jaccard coefficient. To perform a cluster analysis, we used the distance matrix of the Jaccard coefficient and Unweighted Pair Group Method with Arithmetic mean (UPGMA). The reliability of each internal branch of the UPGMA dendrogram was estimated by bootstrap analysis with 1,000 replicates (Phyltools and Phylip packages). Analysis of molecular variance (AMOVA) based on euclidian pairwise distances (Φ*_ST_*) was used to calculate variance component among groups, among strains within groups, and among strains using Arlequin Version 3.0 [56]. The significance of the covariance components associated with the different possible levels of genetic structure was tested using non-parametric permutation procedures (16,000 permutations).

## Supporting Information

Figure S1 Agarose gel electrophoresis of the cry1, cyt1, cyt2, cry4a, cry4b, cry8, cry10, cry11-genes. PCR products obtained with the toxic bacteria isolated from decomposed leaves. M: molecular weight marker.
(0.17 MB DOC)

Table S1 Description of the strains used for AFLP genotyping. All natural worldwide strains were obtained from Pasteur Institute collection.
(0.06 MB DOC)
