# Supplementary material for: Long Lasting Persistence of Bacillus thuringiensis Subsp. israelensis (Bti) in Mosquito Natural Habitats
Source: PLoS One. 2008 Oct 20;3(10):e3432. doi: 10.1371/journal.pone.0003432 (PMC2563433; doi:10.1371/journal.pone.0003432)
Supplement: Figure S1 — Agarose gel electrophoresis of the cry1, cyt1, cyt2, cry4a, cry4b, cry8, cry10, cry11-genes. PCR products obtained with the toxic bacteria isolated from decomposed leaves. M: molecular weight marker. (0.17 MB DOC) [file pone.0003432.s001.doc]

**Figure S1.** Agarose gel electrophoresis of the *cry1*, *cyt1*, *cyt2*, *cry4a*, *cry4b*, *cry8*, *cry10*, *cry11*-gene PCR products obtained with the toxic bacteria isolated from decomposed leaves. M: molecular weight marker.


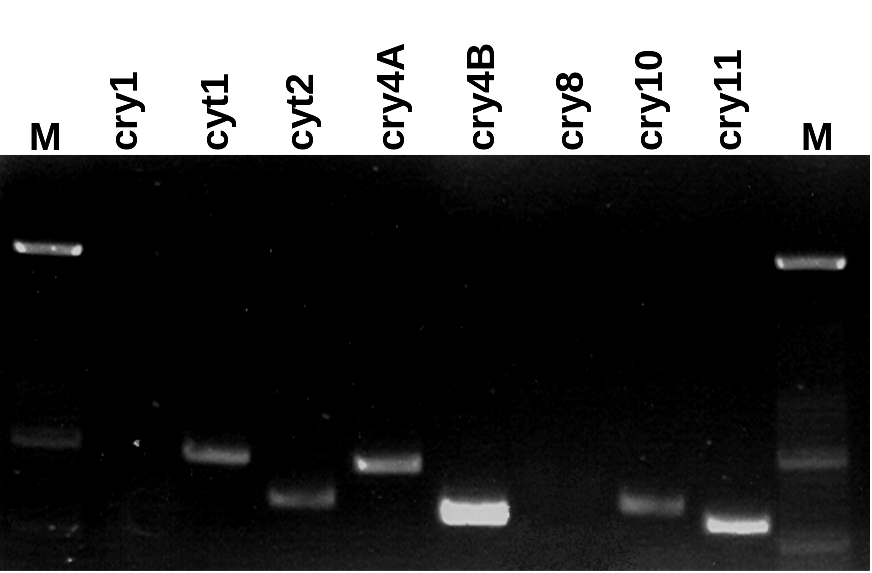


**500 bp**
