# Supplementary material for: Long Lasting Persistence of Bacillus thuringiensis Subsp. israelensis (Bti) in Mosquito Natural Habitats
Source: PLoS One. 2008 Oct 20;3(10):e3432. doi: 10.1371/journal.pone.0003432 (PMC2563433; doi:10.1371/journal.pone.0003432)
Supplement: Table S1 — Description of the strains used for AFLP genotyping. All natural worldwide strains were obtained from Pasteur Institute collection. (0.06 MB DOC) [file pone.0003432.s002.doc]

**Table S1:** Description of the strains used for AFLP genotyping. All natural worldwide strains were obtained from Pasteur Institute collection.
